# Genetic Spectrum of Hemoglobinopathies in Reproductive-Age Individuals from a Hospital-Based Cohort in Guangdong, China: A 7-Year Retrospective Analysis

**DOI:** 10.3390/biomedicines14061326

**Published:** 2026-06-11

**Authors:** Yanchao Wang, Jiajia Xian, Jianchun He, Shaoying Li, Zhenlan Xia, Ding Wang

**Affiliations:** 1 Department of Obstetrics and Gynecology, The Third Affiliated Hospital, Guangzhou Medical University, Guangzhou 510150, China; wycsusan12@163.com (Y.W.); yukixian@126.com (J.X.); zsline123456@163.com (J.H.); amyleewang@163.com (S.L.); 2 Guangdong Provincial Key Laboratory of Major Obstetric Diseases, Guangzhou 510150, China; 3 Guangdong Provincial Clinical Research Center for Obstetrics and Gynecology, Guangzhou 510150, China; 4 Guangdong-Hong Kong-Macao Greater Bay Area Higer Education Joint Laboratory of Maternal-Fetal Medicine, Guangzhou 510150, China; 5 Medical Genetics Center, The Third Affiliated Hospital, Guangzhou Medical University, Guangzhou 510150, China; 6 Department of Orthopedic Surgery, The Third Affiliated Hospital, Guangzhou Medical University, Guangzhou 510150, China; 7 Department of Nursing, The Third Affiliated Hospital, Guangzhou Medical University, Guangzhou 510150, China

**Keywords:** genetic epidemiology, hemoglobinopathies, reproductive-age population, triple-method parallel detection, genotype–phenotype concordance assessment

## Abstract

**Background**: Hemoglobinopathies, including thalassemia and structural hemoglobin variants, are among the most prevalent inherited disorders worldwide and represent a major public health concern in southern China. Accurate characterization of both common and rare variants is essential for carrier screening, genetic counseling, and prevention. However, routine molecular screening is generally restricted to common pathogenic variants, potentially overlooking rare hemoglobinopathy subtypes. This study aimed to characterize the spectrum of hemoglobinopathies in a large hospital-based cohort of reproductive-age individuals from Guangdong, China, and to evaluate a genotype–phenotype discordance-guided secondary testing strategy. **Methods**: A retrospective hospital-based study was conducted in 71,676 reproductive-age individuals who underwent hemoglobinopathy screening at our hospital in Guangdong, China, between 2018 and 2024. Hematological and routine genetic analyses were performed in parallel. Cases exhibiting genotype–phenotype discordance were further investigated using tailored secondary molecular approaches selected according to specific hematological findings. **Results**: In this cohort, 10,412 hemoglobinopathies were identified. Thalassemia accounted for 10,217 cases (98.13%), including α-thalassemia (8026), β-thalassemia (2561), δβ-thalassemia (17), γ-thalassemia (10), and δ-thalassemia (4). Structural hemoglobin variants comprised 195 cases (1.91%). Among the 788 genotype–phenotype discordant cases, secondary analysis yielded a positive detection rate of 36.80% (290/788). **Conclusions**: This study provides large-scale hospital-based data on the distribution of hemoglobinopathies among reproductive-age individuals in Guangdong. Review of genotype–phenotype discordance improved the detection of rare variants beyond routine screening and may facilitate the development of tailored secondary testing strategies. Further studies are warranted to validate its clinical utility and applicability.

## 1. Introduction

Hemoglobinopathies, encompassing both thalassemia and structural hemoglobin variants, represent one of the most common groups of inherited monogenic disorders worldwide and constitute a major public health burden in many tropical and subtropical regions [1,2,3,4]. Structural hemoglobin variants are caused by mutations that lead to an abnormal hemoglobin structure, whereas thalassemia is characterized by the diminished or absent production of specific globin chains that form hemoglobin. On the other hand, variants of thalassemia are classified according to which globin chain production is impaired, leading to varying degrees of anemia. Considering the affected gene/globin chain, thalassemia (Thal) can be classified into *α*-, *β*-, *δ*-, *γ*-, *δβ*-, *γδβ*- and *εγδβ*-thalassemia [2,5,6,7,8]. The geographic and genetic distribution of thalassemia alleles exhibits significant variation across different populations and ethnicities [9,10,11]. These epidemiological differences have important implications for carrier screening strategies, genetic counseling, and regional public health planning.

Compared with Africa, the Mediterranean region, and the Middle East, the hemoglobinopathy spectrum in southern China is characterized by a predominance of α-thalassemia and a distinct distribution of pathogenic variants [9,12,13,14,15,16]. Less common thalassemia variants include *γ*-, *δ*-, *δβ*-, *γδβ*-, and *εγδβ*-thalassemia [7,17,18,19,20]. Despite their rarity, the clinical manifestations of these subtypes can vary depending on the severity of globin chain imbalance. Although uncommon, these variants may present diagnostic challenges and remain important for genetic counseling.

Advances in molecular diagnostic technologies, particularly next-generation sequencing (NGS) and third-generation sequencing (TGS), have facilitated the identification of an increasing number of rare hemoglobinopathy variants [21,22,23,24]. Nevertheless, updated large-scale datasets describing the distribution of common and rare hemoglobinopathy variants in individuals undergoing reproductive screening in Guangdong remain limited.

With the expansion of carrier screening, prenatal diagnosis, and preimplantation genetic testing (PGT), the incidence of severe thalassemia has declined substantially in many high-prevalence regions [1,25]. These interventions may also influence the distribution of hemoglobinopathy variants over time, highlighting the need for updated epidemiological data. Comprehensive characterization of the prevalence and molecular spectrum of hemoglobinopathies remains particularly important in high-prevalence regions such as Guangdong, Southern China [26,27].

However, routine genetic testing in China is largely limited to common thalassemia variants because of cost and technical constraints, potentially leading to under-recognition of rare thalassemia subtypes and structural hemoglobin variants [28,29]. Therefore, strategies that facilitate the identification of cases requiring further molecular investigation may improve the detection of clinically relevant rare variants and complement routine screening programs.

Accordingly, this study aimed to characterize the prevalence and molecular spectrum of hemoglobinopathies in a large hospital-based cohort from Guangdong, Southern China, and to evaluate the utility of genotype–phenotype discordance-guided secondary testing for the detection of rare thalassemia variants and structural hemoglobinopathies.

## 2. Materials and Methods

### 2.1. Sample Collection

This retrospective study was conducted at the Reproductive Medicine Center of our hospital between January 2018 and December 2024. The center is one of the largest reproductive medicine facilities in Guangdong Province, with a high volume of outpatient visits and assisted reproductive treatment cycles.

Individuals of reproductive age who underwent routine hemoglobinopathy screening during the study period were eligible for inclusion. To avoid duplicate enrollment, only the first screening record for each individual was included in the analysis. Repeated visits and duplicate laboratory examinations from the same individual were excluded. When both members of a couple underwent screening, each individual was considered an independent study participant and analyzed separately.

### 2.2. Ethics Approval and Consent to Participate

This study was approved by the Ethics Committee of the Third Affiliated Hospital of Guangzhou Medical University (2024-No. 090) and the need for informed consent for this study was waived by the Ethics Committee of the Third Affiliated Hospital of Guangzhou Medical University.

### 2.3. Hematological Analysis

We collected and analyzed peripheral blood samples from 71,676 patients of reproductive age with fertility needs who visited the Reproductive Medicine Center of our hospital. Neither did the subjects have genetic relationships, nor had any of the patients previously undergone thalassemia genetic testing. Samples underwent routine blood test on hematology automation (Sysmex XN-9000, Shanghai, China) and hemoglobin electrophoresis assays (Capillarys 2 Flex Piercing, Sebia, Evry, France) according to the manufacturer’s instructions. We concurrently evaluated their complete blood count (CBC) parameters, thalassemia screening tests, and conventional α- and β-thalassemia genetic testing profiles.

### 2.4. Molecular Detection of Common α-Thalassemia and β-Thalassemia

Genomic DNA was extracted from peripheral blood samples using a nucleic acid extraction kit (Qiagen, Düsseldorf, Germany). A commercially available α/β-thalassemia gene detection kit (Yaneng Biosciences, Shenzhen, China) was used to identify common α- and β-thalassemia variants in the Chinese population. Gap-PCR was used for 3 α-globin gene deletions (-SEA, -α 3.7, and -α 4.2). Meanwhile, PCR-reverse dot blot (RDB) was used for 3 non-deletional mutations of α-thalassemia [α ConstantSpring (αCS), α QuongSze (αQS), and αWestmead (αWS)] and 17 non-deletional mutations β-thalassemia [CD41/42 (-TCTT), IVS-II-654 (C > T), −28 (A > G), −29 (A > G), −30 (T > C), −32 (C > A), CD14/15 (+G), CD17 (A > T), CD26 (G > A), CD27/28 (+C), CD31 (−C), CD43 (G > T), CD71/72 (+A), IVS-I-1 (G > T), IVS-I-5 (G > C), 5′-UTR Cap+40-43 (del AAAC), Initiation codon mutation (ATG > AGG)]. The assay was performed according to the manufacturer’s instructions.

### 2.5. Definition of Genotype–Phenotype Discordance and Secondary Testing Strategy

Genotype–phenotype discordance was defined as inconsistency between hematological findings and the results of routine molecular screening for common hemoglobinopathy variants. Discordant cases included: (1) hematological abnormalities not explained by the detected genotype; (2) abnormal hematological findings despite negative routine genetic screening; and (3) hemoglobin fractions inconsistent with the expected phenotype of the identified genotype. Such cases were subjected to secondary molecular analysis. Detailed criteria are summarized in (Table 1).

Cases exhibiting genotype–phenotype discordance were subjected to secondary molecular investigations. Secondary testing was performed according to a standardized stepwise workflow guided by hematological findings and routine genetic screening results. Targeted assays with the highest expected diagnostic yield for the suspected abnormality, including Gap-PCR, Sanger sequencing, and MLPA, were preferentially employed as first-line investigations. Third-generation sequencing (TGS) was reserved for cases that remained unresolved after conventional testing or when rare or complex structural variants were suspected.

### 2.6. Secondary Analysis: Analysis of Rare Hemoglobinopathy Genotypes

These strategies integrated multiple methodologies, including Sanger sequencing, specific primer gap-PCR, multiplex ligation-dependent probe amplification (MLPA), and third-generation sequencing (TGS), to ensure accurate and comprehensive genotypic characterization. For individuals suspected of having rare thalassemia subtypes and structural hemoglobin variants that undetectable using common commercial kits, further targeted analyses—such as allele-specific gap-PCR, MLPA, PCR-based Sanger sequencing and TGS—were conducted based on their distinct hematological characteristics. Specific primer Gap-PCR was purchased from Yaneng Biosciences (Shenzhen, Guangdong, China) to detect 6 kinds of *α*-globin gene rare deletions (*αα*HK, -THAI, fusion gene, αααanti3.7, αααanti4.2 and -α27.6) and 3 kinds of β-globin gene rare deletions (Chinese *(Aγδβ)0* thalassaemia, −27 kb of the Southeast Asian (SEA) deletion and Taiwanese deletion). Multiplex ligation-dependent probe amplification (MLPA, MRC Holland; Amsterdam, Netherlands) was conducted to validate deletional fragments of the α-globin and β-globin genes (SALSA^®^ MLPA^®^ Probemix P140-C1 HBA and P102-D1 *HBB*). The detection results were analyzed using Coffalyser.Net software (version 240129.1959; MRC Holland; Amsterdam, The Netherlands). The full-length α- and β-globin genes were amplified, and the purified PCR products were subjected to direct sequencing with an ABI 3500 xl dx genetic analyzer (Applied Biosystems, Foster City, CA, USA). Third-generation sequencing (TGS) was performed using the Sequel II TGS system (Pacific Biosciences Inc., Menlo Park, CA, USA). In cases where conventional diagnostic methods (e.g., Gap-PCR, MLPA) failed to yield conclusive diagnoses for patients with suspected rare or novel thalassemia genotypes, TGS was ultimately employed as a definitive diagnostic approach. Employing single-molecule real-time (SMRT) technology, TGS provided unparalleled resolution in delineating complex structural variants and novel mutations, thereby resolving diagnostic ambiguities that persisted with standard methodologies [28].

### 2.7. Statistical Analysis

The data were entered and managed using Microsoft Excel 2021. The gene mutations, allele frequencies, genotypes, constituent ratios, and spectra of thalassemia and hemoglobinopathy were analyzed using a descriptive method.

## 3. Results

### 3.1. Comprehensive Demographic and Geographic Profiling

**Figure 1:**
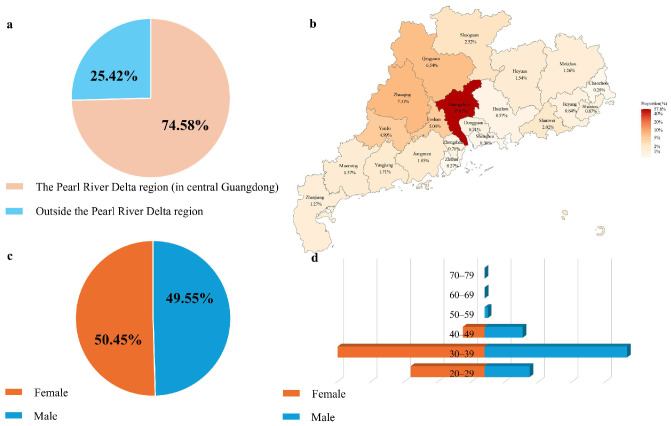
Geographic Distribution and Demographic Characteristics of Reproductive-Age Individuals in a Hospital-Based Cohort from Guangdong, China. (**a**) Constitution of regions. (**b**) Geographic Heat Map. (**c**) Gender Structure Diagram. (**d**) Age Distribution by Gender.

A total of 71,676 peripheral blood samples are from twenty-one cities in Guangdong Province. As is known to all, the Pearl River Delta (PRD) is a dynamic and economically vital region in Guangdong Province, which encompasses nine major cities, including Guangzhou, Shenzhen, and Dongguan, serving as a key hub for trade, manufacturing, and innovation. In total, the proportion of samples comes from the PRD accounts for 74.58%, while areas outside the PRD account for 25.42% (Figure 1a). In the present study, the proportion of each region is: Guangzhou 57.81% (41,402/71,676), Zhaoqing 7.31% (5237/71,676), Qingyuan 6.54% (4685/71,676), Foshan 5.04% (3607/71,676), Yunfu 4.99% (3575/71,676), Shaoguan 2.52% (1807/71,676), Shanwei 2.02% (1450/71,676), Jiangmen 1.85% (1324/71,676), Yangjiang 171% (1225/71,676), Maoming 1.57% (1124/71,676), Heyuan 1.54% (1103/71,676), Zhanjiang 1.27% (906/71,676), Meizhou 1.26% (902/71,676), Shantou 0.87% (622/71,676), Jieyang 0.84% (605/71,676), Dongguan 0.71% (510/71,676), Zhongshan 0.71% (508/71,676), Huizhou 0.57% (409/71,676), Shenzhen 0.30% (215/71,676), Chaozhou 0.28% (204/71,676), Zhuhai 0.27% (196/71,676) (Figure 1b).

### 3.2. Constitution of Gender and Age in Our Population-Based Study

In our study, the gender distribution of the samples is as follows: 49; 54% (35,514/71,676) female and 50.45% (36,162/71,676) male (Figure 1c). We divided ages into six different intervals. Among males, 18.88% (6705/35,514) were in the 20–29 age interval, 63.69% (22,620/35,514) were in the 30–39 age interval, 16.01% (5685/35,514) were in the 40–49 age interval, 1.35% (479/35,514) were in the 50–59 age interval, 0.07% (24/35,514) were in the 60–69 age interval, and one individual was in the 70–79 age interval. Among females, 30.45% (11,012/36,162) were in the 20–29 age interval, 60.61% (21,918/36,162) were in the 30–39 age interval, 8.94% (3232/36,162) were in the 40–49 age interval (Figure 1d).

### 3.3. Prevalence of Hemoglobinopathies in the Study Cohort

Among the 71,676 reproductive-age individuals included in this study, 10,412 (14.53%) were diagnosed with hemoglobinopathies. Thalassemia accounted for 98.13% (10,217/10,412) of cases, whereas structural hemoglobin variants accounted for 1.87% (195/10,412).

**Figure 2:**
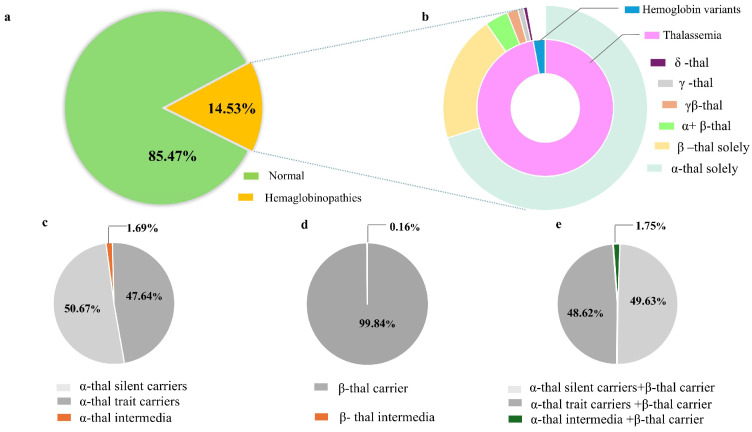
Prevalence and Distribution of Hemoglobinopathies in a Hospital-Based Cohort of Reproductive-Age Individuals from Guangdong, China. (**a**) The Epidemiology of Hemoglobinopathies in Reproductive-Age Individuals from Guangdong. (**b**) Constitution of Hemoglobinopathies in Reproductive-Age Individuals from Guangdong. (**c**) Proportion of individuals with silent, trait, and intermediate *α*-thalassemia in Reproductive-Age Individuals from Guangdong. (**d**) Proportion of β-thalassemia carriers and intermediate cases in Reproductive-Age Individuals from Guangdong. (**e**) Proportion of individuals with Co-inherited *α*- and *β*-thalassemia in Reproductive-Age Individuals from Guangdong.

Among the 10,217 individuals with thalassemia, 8026 carried *α*-thalassemia variants, 2561 carried *β*-thalassemia variants, and 401 carried both *α*- and *β*-thalassemia variants. Because co-inherited α/β-thalassemia cases were included in both *α*- and *β*-thalassemia categories, these groups were not mutually exclusive. In addition, 17 individuals were diagnosed with *δβ*-thalassemia, 10 with *γ*-thalassemia, and 4 with *δ*-thalassemia (Figure 2a,b).

Among the study cohort, 8026 individuals (11.19%) were diagnosed with *α*-thalassemia, including 50.7% silent carriers, 47.6% trait carriers, and 1.7% Hb H disease. Separately, 2561 individuals (3.57%) were diagnosed with β-thalassemia, of whom 99.84% were carriers and 0.16% had *β*-thalassemia intermedia (Figure 2c–e).

### 3.4. Detection of Additional Hemoglobinopathy Variants Through Genotype–Phenotype Discordance Analysis

**Figure 3:**
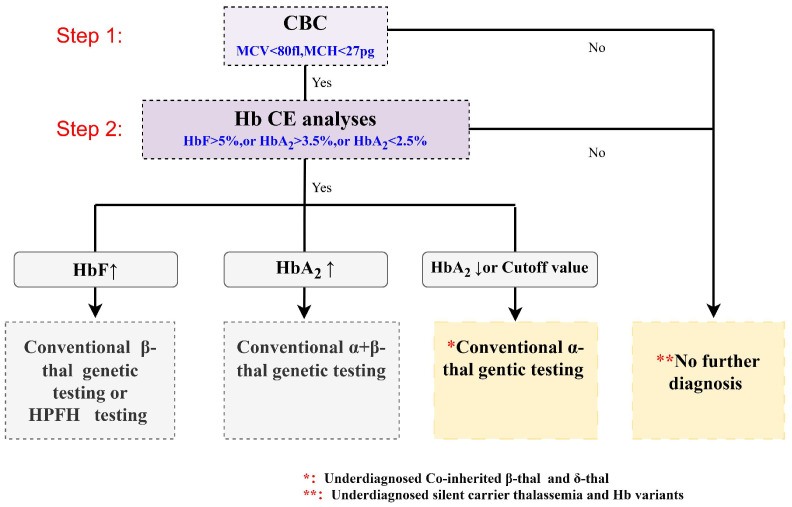
The conventional diagnostic workflow and strategy for thalassemia.

**Figure 4:**
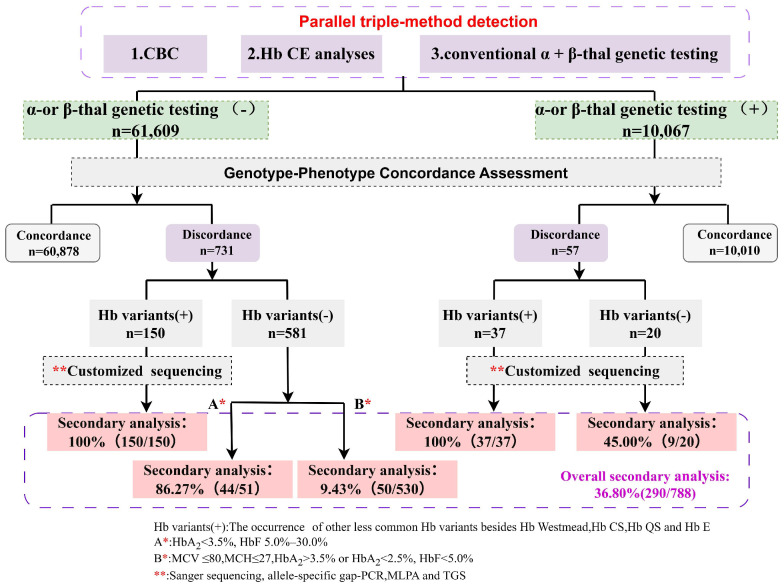
Novel Parallel Diagnostic Workflow and Overall Detection Rate of Thalassemia.

The conventional diagnostic workflow and strategy for thalassemia typically follows a sequential approach (Figure 3). While this stepwise strategy offers cost-effectiveness for population-level screening programs, its critical limitation lies in systematically underdiagnosis of silent α-thalassemia carriers and structural hemoglobinopathies presenting with normal erythrocyte indices (normal MCV and MCH); in the meantime, this strategy may also miss the diagnosis of co-inherited *β*-thalassemia and *δ*-thalassemia in cases where HbA2 is not elevated. To overcome these limitations, we implemented a parallel triple-method integrating hematological, electrophoretic and conventional *α+β* thalassemia molecular analyses (Figure 4).

#### 3.4.1. Novel Parallel Diagnostic Workflow and Overall Detection Rate

Among the 71,676 screened individuals, 788 samples exhibited genotype–phenotype discordance and underwent secondary analysis. Additional pathogenic variants were identified in 290 cases, corresponding to an overall positive detection rate of 36.80% (Table 2, Figure 3).

#### 3.4.2. Secondary Findings in Discordant Cases with Positive Routine Screening Results

Among the 10,067 samples with detected *α*- and/or *β*-thalassemia variants, 57 showed genotype–phenotype discordance. A total of 37 of these cases exhibited abnormal hemoglobin variants, all of which were confirmed by secondary testing (100%, 37/37). Among the remaining 20 discordant cases without abnormal hemoglobin variants, secondary analysis identified additional causative variants in 9 cases (45.00%, 9/20) (Figure 4, Table 3).

#### 3.4.3. Secondary Findings in Discordant Cases with Negative Routine Screening Results

Among the 61,609 samples negative for routine *α*/*β*-thalassemia screening, 731 exhibited genotype–phenotype discordance. Of these, 150 exhibited abnormal hemoglobin variants, all of which were subsequently confirmed by secondary testing (100%, 150/150).

Among the remaining 581 discordant cases without abnormal hemoglobin variants, the diagnostic yield differed substantially between hematological subgroups. Secondary testing identified causative variants in 86.27% (44/51) of individuals in Subgroup A (HbA_2_ < 3.5% and HbF 5–30%), compared with 9.43% (50/530) of individuals in Subgroup B (HbA_2_ > 3.5% or HbA_2_ < 2.5% without HbF elevation) (Table 4 and Table S1).

#### 3.4.4. Representative Rare and Novel Variants Identified

**Figure 5:**
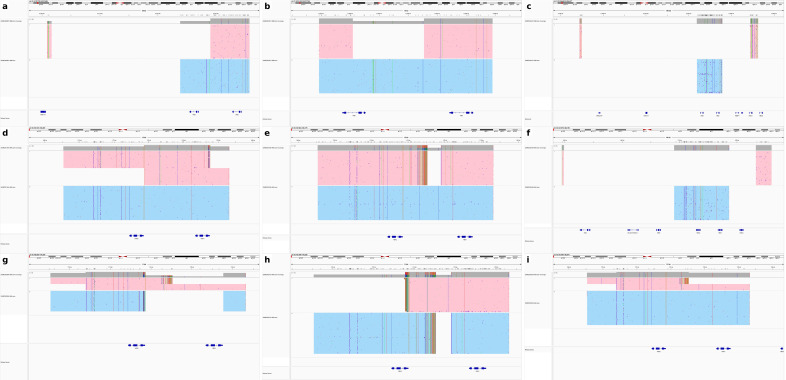
Schematic Diagram Illustrating the Resolution of Complex Thalassemia Genomic Variations via TGS. (**a**) β(SEA-HPFH)/βN. (**b**) chr11:5226187-5231090 deletion (4.903Kb)/βN. (**c**) Gγ(Aγδβ)0/βN. (**d**) αααanti3.7/αα. (**e**) αα/ααHK. (**f**) -THAI/αα. (**g**) αααanti4.2/-α3.7. (**h**) ααHK/-α4.2. (**i**) αααanti4.2/αα.

Secondary molecular investigations identified a broad spectrum of rare thalassemia variants and structural hemoglobinopathies that were not identified by routine screening approaches. Complex genomic rearrangements were characterized using MLPA and third-generation sequencing (TGS) (Figure 5). A previously reported 4.903 kb *HBB* deletion (chr11:5226187–5231090), first described in an individual from Shenzhen, Guangdong Province, was also identified in the present cohort (Figure 5).

### 3.5. Genotypic Spectrum of Hemoglobinopathies

#### 3.5.1. Genotype Spectrum of *α*-Thalassemia

A total of 38 *α*-thalassemia genotypes were identified among 8026 individuals with α-thalassemia. Conventional genotypes accounted for 98.98% (7944/8026) of cases, whereas rare genotypes requiring secondary molecular confirmation accounted for 1.02% (82/8026). The genotype distribution was dominated by six common genotypes: -SEA/αα (46.46%), -α3.7/αα (27.12%), -α4.2/αα (10.67%), ααWS/αα (7.84%), ααCS/αα (2.78%), and ααQS/αα (1.66%), which together represented 96.52% of all α-thalassemia cases. Detailed distributions of all 38 genotypes are provided in Supplementary Materials Table S2.

#### 3.5.2. Genotype Spectrum of *β*-Thalassemia

A total of 29 *β*-thalassemia genotypes were identified among 2561 individuals with *β*-thalassemia. Conventional genotypes detected by routine commercial assays accounted for 98.48% (2522/2561) of cases, whereas rare genotypes requiring secondary molecular confirmation accounted for 1.52% (39/2561). The genotype distribution was dominated by nine common genotypes: βCD41-42/βN (39.87%), βIVS-II-654/βN (22.73%), β-28/βN (15.23%), βCD17/βN (8.40%), βCD26/βN (3.98%), βCD71-72/βN (2.30%), βcap/βN (1.41%), β-29/βN (1.02%), and βCD27-28/βN (0.98%), which together represented 95.90% of all β-thalassemia cases. Detailed distributions of all 29 genotypes are provided in Supplementary Materials Table S3.

#### 3.5.3. Genotype Spectra of Co-Inherited *α*- and *β*-Thalassemia

A total of 54 distinct genotype combinations were identified among individuals with co-inherited α- and β-thalassemia variants. The five most common combinations were αα/-SEA with βCD41-42/βN (19.70%), αα/-SEA with βIVS-II-654/βN (11.97%), αα/-α3.7 with βCD41-42/βN (10.72%), αα/-SEA with β-28/βN (7.48%), and αα/-α3.7 with βIVS-II-654/βN (5.49%). Detailed distributions of all 54 genotype combinations are provided in Supplementary Materials Table S4.

#### 3.5.4. Genotype Spectra of *δβ*-, *γ*-, and *δ*-Thalassemia

A total of 17 individuals were identified with *δβ*-thalassemia, all carrying the Chinese (Aγδβ)^0^ deletion. In addition, 10 individuals with *γ*-thalassemia carried five distinct variants, among which γ-196 C > T and Aγ (+25 G > A) were the most frequently observed. A total of 4 individuals with δ-thalassemia carried three distinct *HBD* variants, including δ-30 T > C, δ-77 T > C, and δCD87(CAG > TAG). Detailed genotype distributions are provided in Supplementary Materials Table S5.

#### 3.5.5. Genotype Spectrum of Structural Hemoglobin Variants

**Figure 6:**
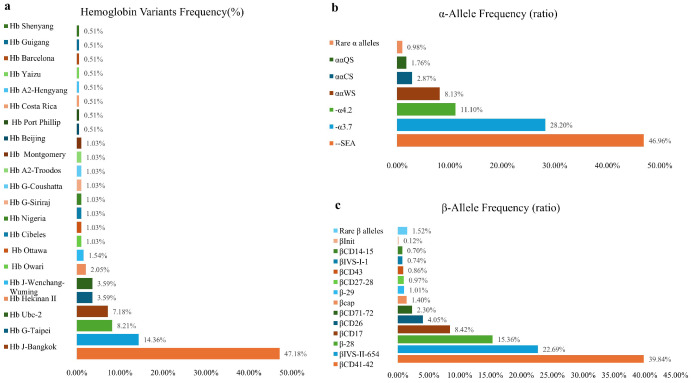
Distribution of Structural Hemoglobin Variants and Thalassemia Alleles in a Hospital-Based Cohort of Reproductive-Age Individuals from Guangdong, China. (**a**) Hemoglobin Variant Frequency; (**b**) *α*-allele frequency; (**c**) *β*-allele frequency.

A total of 195 individuals carried structural hemoglobin variants, representing 24 distinct genotypes involving *HBA1*, *HBA2*, *HBB*, and *HBD* genes. Hb New York (47.18%), Hb Q-Thailand (14.36%), Hb G-Honolulu (8.21%), Hb J-Bangkok (7.18%), Hb G-Taipei (3.59%), and Hb Ube-2 (3.59%) were the most prevalent variants. The remaining variants individually accounted for less than 2% of cases. Detailed genotype distributions are provided in Supplementary Materials Table S6 and Figure 6a.

### 3.6. Distribution of Pathogenic α- and β-Thalassemia Alleles

Allele-frequency analysis demonstrated that the six most prevalent α-thalassemia alleles (-SEA, -α3.7, -α4.2, ααWS, ααCS, and ααQS) accounted for 99.02% of all pathogenic α-globin alleles identified in this cohort (Supplementary Materials Table S7 and Figure 6b).

Similarly, the thirteen most prevalent *β*-thalassemia alleles collectively accounted for 98.48% of all pathogenic *β*-globin alleles identified in this cohort, with βCD41-42, βIVS-II-654, and β-28 representing the three most common alleles (Supplementary Materials Table S8 and Figure 6c).

## 4. Discussion

This study represents a large-scale hospital-based retrospective analysis of hemoglobinopathies among reproductive-age individuals attending a reproductive medicine center in Guangdong, Southern China.

Regarding Cohort Characteristics and Potential Selection Bias

The composition of the study cohort is an important consideration when interpreting the present findings. Unlike newborn-screening cohorts, antenatal population-based cohorts, or community-based surveys, this study was conducted in a single-center reproductive medicine setting. Therefore, potential selection bias cannot be excluded when interpreting the findings [30,31]. Nevertheless, this cohort remains clinically relevant because it comprises individuals undergoing routine hemoglobinopathy screening during their reproductive years, the period most directly associated with reproductive risk assessment and carrier screening. In addition, our institution receives a large number of patients from different regions of Guangdong Province, providing a valuable opportunity to characterize the spectrum of common and rare hemoglobinopathy variants encountered in clinical practice. Therefore, the findings should be interpreted as large-scale hospital-based data rather than estimates of population-level prevalence in Guangdong.

Regarding Diagnostic Workflow and Strategy

Conventional thalassemia screening strategies typically rely on abnormal hematological findings to trigger subsequent genetic testing. Although this approach is cost-effective for large-scale screening programs, it may fail to identify certain clinically relevant findings, including silent α-thalassemia carriers with normal hematological indices and some structural hemoglobin variants [32,33]. In addition, sequential testing may prolong the diagnostic process in selected cases.

To address these limitations, we incorporated genotype–phenotype concordance assessment into a parallel screening strategy combining hematological analysis, hemoglobin testing, and routine *α*/*β*-thalassemia genetic testing. Secondary molecular investigations, including Gap-PCR, Sanger sequencing, MLPA, and TGS, were performed when clinically indicated according to the discordant phenotype.

This integrated approach may offer several diagnostic advantages, including improved detection of α-silent carriers and structural hemoglobin variants that may be missed by conventional screening strategies. Compared with a previous large-scale study from Fujian [34], a substantially broader spectrum of structural hemoglobin variants was identified in our cohort. To our knowledge, few previous large-scale hemoglobinopathy studies have systematically incorporated genotype–phenotype discordance-guided secondary testing into their screening workflows [34,35].

Using this approach, 4064 α-silent carriers were identified, many of whom may not have been identified by hematology-based screening alone. Furthermore, secondary testing identified additional pathogenic variants in 36.8% (290/788) of discordant cases. The diagnostic yield was particularly high among individuals with abnormal hemoglobin variants and among those with elevated HbF levels (HbA_2_ < 3.5% and HbF 5–30%), suggesting that genotype–phenotype discordance may serve as a useful indicator for further molecular investigation. However, these findings were derived from a retrospective single-center cohort and require validation in prospective multicenter studies before broader implementation can be recommended.

Regarding the Application of Novel Methodologies

Recent advances in next-generation sequencing (NGS) and third-generation sequencing (TGS) have substantially improved the detection of rare and complex hemoglobinopathy variants [21,24,36,37,38]. However, their routine use as first-line screening tools remains limited by cost, technical complexity, and the need for further standardization. Consequently, conventional hematological screening and targeted genetic testing continue to serve as the foundation of current diagnostic practice.

In the present study, advanced molecular technologies were not used as universal screening tools but were selectively applied to cases exhibiting genotype–phenotype discordance. This strategy enabled targeted investigation of unresolved cases and facilitated the identification of rare thalassemia variants and structural hemoglobinopathies that would not have been detected by routine testing alone. Our findings therefore support a complementary role for NGS and TGS within a stepwise diagnostic framework guided by hematological findings, routine genetic testing, and genotype–phenotype concordance assessment.

Compared with approaches that rely solely on conventional mutation panels for epidemiological investigations, this workflow may improve the detection of rare variants while maintaining the practicality of routine screening. Nevertheless, further prospective multicenter studies are required to evaluate its clinical utility, cost-effectiveness, and applicability in broader screening settings.

Study Limitations

Several limitations should be acknowledged. First, secondary molecular investigations were performed only in selected genotype–phenotype discordant cases rather than across the entire cohort. Second, the clinical utility and cost-effectiveness of the proposed workflow require further evaluation in prospective multicenter studies.

Taken together, this study provides large-scale hospital-based data on the genetic spectrum of hemoglobinopathies among reproductive-age individuals in Guangdong, Southern China. In addition to characterizing the distribution of common variants, our findings highlight the contribution of genotype–phenotype discordance-guided secondary testing to the detection of rare thalassemia variants and structural hemoglobinopathies that may be missed by routine screening approaches. These results support further investigation of tailored secondary testing strategies and provide a foundation for future multicenter studies aimed at optimizing hemoglobinopathy screening and diagnostic workflows.

## 5. Conclusions

This study provides large-scale hospital-based data on the genetic spectrum of hemoglobinopathies among reproductive-age individuals in Guangdong, Southern China. In addition to characterizing the distribution of common hemoglobinopathy variants, our findings demonstrate that genotype–phenotype discordance-guided secondary testing can improve the detection of rare thalassemia variants and structural hemoglobinopathies that may not be identified by routine screening alone. These findings expand current knowledge of hemoglobinopathy variation in this population and support further validation of tailored secondary testing strategies in broader clinical settings.

## Figures and Tables

**Table 1:** Criteria for genotype–phenotype discordance and indications for secondary testing.

Category	Discordant Findings	Potential Explanation	Secondary Testing Methods
a	Microcytosis and/or hypochromia inconsistent with routine genetic screening results	Rare *α*- or *β*-thalassemia variants not covered by routine assays	Gap-PCR, MLPA, Sanger sequencing, or TGS
b	Elevated HbA2 without detectable *β*-thalassemia variants	Rare *β*-globin gene defects	*HBB* sequencing or TGS
c	Reduced or unexpectedly normal HbA2 levels, particularly in individuals carrying *β*-thalassemia variants	Co-inherited *δ*-thalassemia or *δ*-globin variants	*HBD* sequencing or TGS
d	Unexplained elevation of HbF levels	*δβ*-thalassemia, HPFH, *γ*-globin defects, or regulatory variants	Gap-PCR, MLPA, or TGS
e	Abnormal hemoglobin fractions or electrophoretic patterns unexplained by routine screening results	Structural hemoglobin variants	Globin gene sequencing or TGS
f	Hematological abnormalities more severe than expected for the identified genotype	Additional pathogenic variants, copy-number variants, or complex genotypes	MLPA, extended molecular analysis, or TGS

Note: Secondary testing methods were selected according to a standardized stepwise workflow. Simpler targeted assays were prioritized, whereas TGS was reserved for unresolved cases or suspected complex structural variants.

**Table 2:** Genotype–phenotype accordance cases and secondary analysis positive rate.

	Genotype–Phenotype Accordance	Genotype–Phenotype Discordance	Secondary Analysis Positive	Secondary Analysis Positive Rate
Initial genetic test positive	10,010	57	46	80.70%
Initial genetic test negative	60,878	731	244	33.38%
Total	70,888	788	290	36.80%

**Table 3:** Secondary Findings of Initial Genetic Testing Positive Samples.

**Case Detected**	**Gender**	**MCV** (fL)	**MCH** (pg)	**HbA2** (%)	**HbF** (%)	**HbVar** (%)	**Conventional Genetic Result**	**Secondary Analysis Finding**
16	F	74.9 ± 2.0	23.23 ± 0.7	1.63 ± 0.1	28.2 ± 0.9	Z1 zone = 0.7–0.9%	αα/-α4.2, βN/βN	*HBA1*: c.223G > C (Hb Q-Thailand)
11	M	78.4 ± 1.8	26.0 ± 0.6	1.76 ± 0.4		Z1 zone = 0.7–0.9%	αα/-α4.2, βN/βN	*HBA1*: c.223G > C (Hb Q-Thailand)
1	F	61.4	20.1	4.4	16.0	Z1 zone 1.2%	αα/-α4.2, βIVS-II-654/βN	*HBA1*: c.223G > C (Hb Q-Thailand)
1	F	68.9	19.7	1.9	15.5	Z10 zone 20.9%; Z1 zone 1.8%	-α4.2/-SEA, βN/βN	*HBA1*: c.223G > C (Hb Q-Thailand)
1	F	68.4	20.7	5.5	-	-	αα/-α3.7, βN/βN	βCD8/9 (+G)/βN
1	M	69	22.9	3.5	19.6	-	αα/^-SEA^, β^N^/β^N^	β^(SEA-HPFH)^/β^N^
1	M	68.1	21.6	2.9	-	Z1 zone 35.8%	αα/^-SEA^, β^N^/β^N^	*HBB*: c.341 T > A (Hb NewYork)
1	F	67	22.1	5.4	3.9	Z11 zone 90.7%	αα/αα, β^CD41−42^/β^N^	*HBB*: c.341 T > A (Hb NewYork)
1	F	77.4	25.1	2.9	-	Z11 zone 39.1%	αα/-α4.2, β^N^/β^N^	*HBB*: c.341 T > A (Hb NewYork)
1	M	84.6	27.9	2.5		Z11 zone 42.2%	αα/-α3.7, β^N^/β^N^	*HBB*: c.341 T > A (Hb NewYork)
1	F	71.6	23	4.4	-	HbD zone 17.4%	αα/αα, β^IVS-II−654^/β^N^	*HBA2*: c.91 G > C (Hb G-Honolulu)
1	F	93.8	31.6	2.1	-	HbD zone 23.5%, Z1 zone0.7%	αα/-α4.2, β^N^/β^N^	*HBA2*: c.91G > C (Hb G-Honolulu)
1	M	60.9	19.5	3.2	-	-	αα/αα, β^IVS-II−654^/β^N^	*HBD*: c.-80T > C
1	M	59.8	19	5.3	-	-	αα/αα^WS^, β^N^/β^N^	β^CD 15 (TGG > TGA)^/β^N^
1	F	74.7	20.5	0.5	-	HbH 25.8%, HbBart’s 1.1%	αα/^-SEA^, β^N^/β^N^	αα^Init CD ATG > AAG^/^-SEA^
1	M	66.5	21.0	4.3	-	HbD 8.2%	αα/^-SEA^, β^N^/β^N^	*HBB*: c.22G > A (Hb G-Siriraj)
1	M	68.2	20.8	2.4		Z12 zone 48.9%	αα/^-SEA^, β^N^/β^N^	*HBB*: c.170G > A (Hb J-Bangkok)
1	M	68.9	21.7	3.5	41.6	-	αα/αα, β^IVS-II−654^/β^N^	γ^−196 C > T^/γ^N^
1	F	68.7	20.4	1.3	12.7	-	αα/^-SEA^, β^N^/β^N^	γ^−196 C > T^/γ^N^
1	F	60.7	16.4	3.5	-	Z8 zone 26.9%	αα/^-SEA^, β^N^/β^N^	*HBA2*: c.275T > C (Hb Port Phillip)
1	M	68.6	20.8	2.8		HbD zone 37%	αα/^-SEA^, β^N^/β^N^	*HBB*: c.68A > C (Hb G-Coushatta)

**Table 4:** Secondary Testing Outcomes in Genotype–Phenotype Discordant Cases Negative for Routine *α*/*β*-Thalassemia Screening.

Subgroup	Hematological Subgroup Definition	No. of Cases	Positive Findings	Diagnostic Yield (%)
A	HbA_2_ < 3.5% and HbF 5–30%	51	44	86.27
B	HbA_2_ > 3.5% or HbA_2_ < 2.5% without HbF elevation	530	50	9.43

Note: The table summarizes secondary testing outcomes among discordant cases without abnormal hemoglobin variants. Individual hematological parameters and secondary findings for Subgroup A are provided in Supplementary Materials Table S1. Subgroup B is summarized at the group level because of the large number of cases and heterogeneous secondary findings.

## Data Availability

The original contributions presented in this study are included in the article/Supplementary Materials. Further inquiries can be directed to the corresponding authors.

## Supplementary Materials

The following supporting information can be downloaded at: https://www.mdpi.com/article/10.3390/biomedicines14061326/s1, Table S1: Hematological Parameters and Secondary Testing Findings in Discordant Cases of Subgroup A. Table S2: Spectrum of *α*-thalassemia mutations among people of reproductive age in Guangdong province, Southern China. Table S3: Spectrum of *β*-thalassemia mutations among people of reproductive age in Guangdong province, Southern China. Table S4: Spectrum of co-inherited α- and *β*-thalassemia among people of reproductive age in Guangdong province, Southern China. Table S5: Spectrum of *δ*- thalassemia, *δβ*- thalassemia and γ-thalassemia among people of reproductive age in Guangdong province, Southern China. Table S6: Spectrum and phenotypes of hemoglobinopathy among people of reproductive age in Guangdong province, Southern China. Table S7: Allele frequency of α-thalassemia mutations among people of reproductive age in Guangdong province, Southern China. Table S8: Allele frequency of *β*-thalassemia mutations among people of reproductive age in Guangdong province, Southern China.

## Abbreviations

The following abbreviations are used in this manuscript:

Thal	Thalassemia
