# Supplementary material for: Genetic Spectrum of Hemoglobinopathies in Reproductive-Age Individuals from a Hospital-Based Cohort in Guangdong, China: A 7-Year Retrospective Analysis
Source: Biomedicines. 2026 Jun 11;14(6):1326. doi: 10.3390/biomedicines14061326 (PMC13296744; doi:10.3390/biomedicines14061326)
Supplement: Supplementary file 1 [file biomedicines-14-01326-s001.zip › biomedicines-4335379-supplementary.pdf]

Supplementary Data Table S1: Hematological Parameters and Secondary Testing Findings in Discordant Cases of Subgroup A

Case detected	gender	MCV(fL)	MCH(pg)	HbA2(%)	HbF(%)	Conventional genetic result	Secondary analysis finding
**2**	F	95.5±2.9	32.3±1.98	1.7±0.2	16.1±1.63	αα/αα, β^N/β^N	$\gamma^{-196\ C>T}/\gamma^N$
**3**	M	83.0±13.5	27.2±5.2	1.73±0.4	14.2±2.18	αα/αα, β^N/β^N	$\gamma^{-196\ C>T}/\gamma^N$
**1**	M	68.7	20.4	1.3	12.7	αα/αα, β^N/β^N	$\gamma^{-219\ (+AGCA)}/\gamma^N$
**1**	F	93.0	31.3	1.8	14.6	αα/αα, β^N/β^N	$\gamma^{-196\ C>T}/\gamma^{A\gamma(+25\ G>A)}$
**1**	F	82.5	21.9	2.3	14.6	αα/αα, β^N/β^N	$\gamma^{-158\ C>T}/\gamma^{A\gamma(+25\ G>A)}$
**7**	M	77.25±4.6	25.1±1.79	3.80±0.65	22.58±3.6	αα/αα, β^N/β^N	$\beta^{(SEA-HPFH)}/\beta^N$
**6**	F	8.45±2.7	26.9±0.8	4.3±0.75	25.7±3.2	αα/αα, β^N/β^N	$\beta^{(SEA-HPFH)}/\beta^N$
**3**	F	68.85±1.9	22.3±0.2	6.4±0.2	6.75±1.7	αα/αα, β^N/β^N	$\beta^{Taiwanese\ deletion}/\beta^N$
**2**	M	68.55±1.7	21.45±0.07	7.45±0.6	5.8±0.7	αα/αα, β^N/β^N	$\beta^{Taiwanese\ deletion}/\beta^N$
**8**	M	73.3±3.2	23.9±2.1	2.7±0.46	16.7±3.5	αα/αα, β^N/β^N	$\beta^{G\gamma+\ (A\gamma\delta\beta)\ 0}/\beta^N$
**9**	F	71.4±3.6	22.8±2.2	2.5±0.55	14.8±4.2	αα/αα, β^N/β^N	$\beta^{G\gamma+\ (A\gamma\delta\beta)\ 0}/\beta^N$
**1**	M	65.8	20.9	7.0	6.2	αα/αα, β^N/β^N	$\beta^{deletion(4.903Kb)}/\beta^N$

Note: Subgroup A was defined as genotype–phenotype discordant cases with negative routine α/β-thalassemia screening, no abnormal hemoglobin variants, $HbA_2 < 3.5\%$, and HbF levels of 5–30%. This table presents individual-level hematological parameters and secondary molecular findings for Subgroup A, which showed a relatively high diagnostic yield. Subgroup B was not listed individually because of the large sample size and heterogeneous findings; its overall diagnostic yield is summarized in Table 4.

Supplementary Data Table S2. Spectrum of α-thalassemia mutations among people of reproductive age in Guangdong province, Southern China.

	**genotype**	**Phenotype**	**cases detected**	**Frequency (%)**
Common α-thalassemia	$\alpha\alpha/--^{SEA}$	α^0/α	3729	46.462
	$\alpha\alpha/-\alpha^{3.7}$	α^+/α	2177	27.124
	$\alpha\alpha/-\alpha^{4.2}$	α^+/α	856	10.665
	$\alpha\alpha/\alpha\alpha^{WS}$	α^+/α	629	7.837
	$\alpha\alpha/\alpha\alpha^{CS}$	α^+/α	223	2.778
	$\alpha\alpha/\alpha\alpha^{QS}$	α^+/α	133	1.657
	$-\alpha^{3.7}/--^{SEA}$	α^+/α^0	69	0.860
	$-\alpha^{4.2}/--^{SEA}$	α^+/α^0	32	0.399
	$\alpha\alpha^{WS}/--^{SEA}$	α^+/α^0	23	0.287
	$-\alpha^{3.7}/-\alpha^{3.7}$	α^+/α^+	20	0.249
	$-\alpha^{3.7}/-\alpha^{4.2}$	α^+/α^+	16	0.199
	$\alpha\alpha^{WS}/-\alpha^{3.7}$	α^+/α^+	10	0.125
	$\alpha\alpha^{QS}/-\alpha^{3.7}$	α^+/α^+	4	0.050
	$\alpha\alpha^{CS}/-\alpha^{3.7}$	α^+/α^+	4	0.050
	$\alpha\alpha^{CS}/--^{SEA}$	α^+/α^0	3	0.037
	$-\alpha^{4.2}/-\alpha^{4.2}$	α^+/α^+	4	0.050
	$\alpha\alpha^{QS}/--^{SEA}$	α^+/α^0	3	0.037
	$\alpha\alpha^{WS}/\alpha\alpha^{WS}$	α^+/α^+	2	0.025
	$\alpha\alpha^{CS}/\alpha\alpha^{CS}$	α^+/α^+	2	0.025
	$\alpha\alpha^{QS}/\alpha\alpha^{QS}$	α^+/α^+	1	0.012
	$\alpha\alpha^{QS}/\alpha\alpha^{CS}$	α^+/α^+	1	0.012
	$\alpha\alpha^{WS}/\alpha\alpha^{CS}$	α^+/α^+	1	0.012
	$\alpha\alpha^{WS}/\alpha\alpha^{QS}$	α^+/α^+	1	0.012
	$\alpha\alpha^{QS}/-\alpha^{4.2}$	α^+/α^+	1	0.012
Partial Sum			7944	98.978
Rare α-thalassemia	$\alpha\alpha/\alpha\alpha^{HK}$	α^+/α^0	43	0.536
	$--^{THAI}/\alpha\alpha$	α^0/α	15	0.187
	$HK\alpha\alpha/--^{SEA}$	α^+/α^0	4	0.050
	$\alpha\alpha\alpha^{anti4.2}/-\alpha^{3.7}$	$\alpha^+/\alpha\alpha\alpha$	2	0.025
	$Fusion/^{--SEA}$	α^+/α^0	1	0.012
	$\alpha\alpha^{HK}/-\alpha^{4.2}$	α^+/α^+	1	0.012
	$\alpha\alpha^{3.7}/\alpha\alpha^{HK}$	α^+/α^+	1	0.012

	$\alpha\alpha^{4.2}/\alpha\alpha^{HK}$	α^{+}/α^{+}	1	0.012
	$\alpha\alpha^{WS}/\alpha\alpha^{HK}$	α^{+}/α^{+}	1	0.012
	$\alpha\alpha\alpha^{anti3.7}$	/	3	0.037
	$\alpha\alpha\alpha^{anti4.2}$	/	2	0.025
	$\alpha\alpha^{CD\ 30\ -GAG}/\alpha\alpha$	α^{+}/α	6	0.075
	$\alpha\alpha^{Init\ CD\ ATG>AAG}/\alpha\alpha$	α^{+}/α	1	0.012
	$\alpha\alpha^{Init\ CD\ ATG>AAG}/--^{SEA}$	α^{+}/α^{0}	1	0.012
Partial Sum			82	1.022
Sum			8026	100

Supplementary Data Table S3. Spectrum of β-thalassemia mutations among people of reproductive age in Guangdong province, Southern China.

	**genotype**	**Phenotype**	**cases detected**	**Frequency (%)**
Common β-thalassemia	$\beta^{CD41-42}/\beta^{N}$	β^{0}/β^{N}	1021	39.867
	$\beta^{IVS-II-654}/\beta^{N}$	β^{+}/β^{N}	582	22.725
	β^{-28}/β^{N}	β^{+}/β^{N}	390	15.228
	β^{CD17}/β^{N}	β^{0}/β^{N}	215	8.395
	β^{CD26}/β^{N}	β^{E}/β^{N}	102	3.983
	$\beta^{CD71-72}/\beta^{N}$	β^{0}/β^{N}	59	2.304
	β^{cap}/β^{N}	β^{+}/β^{N}	36	1.406
	β^{-29}/β^{N}	β^{+}/β^{N}	26	1.015
	$\beta^{CD27-28}/\beta^{N}$	β^{0}/β^{N}	25	0.976
	β^{CD43}/β^{N}	β^{0}/β^{N}	22	0.859
	$\beta^{CD14-15}/\beta^{N}$	β^{0}/β^{N}	19	0.703
	$\beta^{IVS-I-1}/\beta^{N}$	β^{0}/β^{N}	18	0.742
	β^{Init}/β^{N}	β^{0}/β^{N}	3	0.117
	β^{E}/β^{E}	β^{+}/β^{+}	1	0.039
	β^{-28}/β^{-28}	β^{+}/β^{+}	1	0.039
	$\beta^{-28}/\beta^{CD41-42}$	β^{+}/β^{0}	1	0.039
	β^{-28}/β^{CD17}	β^{+}/β^{0}	1	0.039
Partial Sum			2522	98.477

Rare β-thalassemia	South East Asian (SEA) deletion）/β^{N}	β^{0}/β^{N}	14	0.547
	Taiwanese deletion/β^{N}	β^{0}/β^{N}	5	0.195
	$\beta^{-90\ (C->T)}/\beta^{N}$	β^{+}/β^{N}	2	0.078
	$\beta^{CD\ 89\text{-}93\ (-14bp)}/\beta^{N}$	β^{0}/β^{N}	2	0.078
	$\beta^{IVS\text{-}I(-2)}/\beta^{N}$	β^{0}/β^{N}	1	0.039
	$\beta^{CD8/9\ (+G)}/\beta^{N}$	β^{0}/β^{N}	1	0.039
	$\beta^{CD\ 43\ (GAG>TAG)}/\beta^{N}$	β^{0}/β^{N}	1	0.039
	$\beta^{CD\ 15\ (TGG>TGA)}/\beta^{N}$	β^{0}/β^{N}	1	0.039
	$\beta^{CD\ 37\ (TGG>TAG)}/\beta^{N}$	β^{0}/β^{N}	6	0.234
	$\beta^{CD\ 19(AAC>AGC)}/\beta^{N}$	β^{+}/β^{N}	1	0.039
	$\beta^{IVS\ II\text{-}654\ C>T}/\beta^{N}$	β^{+}/β^{N}	1	0.039
	$\beta^{IVS\text{-}I\text{-}110}/\beta^{N}$	β^{+}/β^{N}	1	0.039
	$\beta^{CD\ 27/28\ (+C)}/\beta^{N}$	β^{0}/β^{N}	1	0.039
	$\beta^{-50\ G>A}/\beta^{N}$	NA	1	0.039
	chr11:5226187-5231090 deletion(4.903Kb)/β^{N}	β^{0}/β^{N}	1	0.039
Partial Sum			39	1.523
Sum			2561	100

Supplementary Data Table S4. Spectrum of co-inherited α- and β-thalassemia among people of reproductive age in Guangdong province, Southern China.

genotype	Phenotype	cases detected	Frequency (%)
$\alpha\alpha/^{--SEA}$, $\beta^{CD41\text{-}42}/\beta^{N}$	α^{0}/α, β^{0}/β^{N}		19.701
$\alpha\alpha/^{--SEA}$, $\beta^{IVS\text{-}II\text{-}654}/\beta^{N}$	α^{0}/α, β^{+}/β^{N}	48	11.970
$\alpha\alpha/\text{-}\alpha^{3.7}$, $\beta^{CD41\text{-}42}/\beta^{N}$	α^{+}/α, β^{0}/β^{N}	43	10.723
$\alpha\alpha/^{--SEA}$, β^{-28}/β^{N}	α^{0}/α, β^{+}/β^{N}	30	7.481
$\alpha\alpha/\text{-}\alpha^{3.7}$, $\beta^{IVS\text{-}II\text{-}654}/\beta^{N}$	α^{+}/α, β^{+}/β^{N}	22	5.486
$\alpha\alpha/\alpha\alpha^{WS}$, $\beta^{CD41\text{-}42}/\beta^{N}$	α^{+}/α, β^{0}/β^{N}	17	4.239
$\alpha\alpha/^{--SEA}$, β^{CD17}/β^{N}	α^{0}/α, β^{0}/β^{N}	18	4.489
$\alpha\alpha/\text{-}\alpha^{3.7}$，$\beta^{-28}/\beta^{N}$	α^{+}/α, β^{+}/β^{N}	14	3.491

$\alpha\alpha/-\alpha^{4.2}$, $\beta^{CD41-42}/\beta^{N}$	α^{+}/α, β^{0}/β^{N}	13	3.242
$\alpha\alpha/^{--SEA}$, β^{CD26}/β^{N}	α^{0}/α, β^{E}/β^{N}	11	2.743
$\alpha\alpha/-\alpha^{4.2}$, β^{-28}/β^{N}	α^{+}/α, β^{+}/β^{N}	8	1.995
$\alpha\alpha/\alpha\alpha^{WS}$, $\beta^{IVS-II-654}/\beta^{N}$	α^{+}/α, β^{+}/β^{N}	8	1.995
$\alpha\alpha/-\alpha^{4.2}$, $\beta^{IVS-II-654}/\beta^{N}$	α^{+}/α, β^{+}/β^{N}	7	1.746
$\alpha\alpha/\alpha\alpha^{CS}$, $\beta^{CD41-42}/\beta^{N}$	α^{+}/α, β^{0}/β^{N}	7	1.746
$\alpha\alpha/-\alpha^{3.7}$, β^{CD26}/β^{N}	α^{+}/α, β^{E}/β^{N}	7	1.746
$\alpha\alpha/-\alpha^{3.7}$, β^{CD17}/β^{N}	α^{+}/α, β^{0}/β^{N}	7	1.746
$\alpha\alpha/-\alpha^{4.2}$, β^{CD17}/β^{N}	α^{+}/α, β^{0}/β^{N}	6	1.496
$\alpha\alpha/-\alpha^{3.7}$, $\beta^{CD27/28}/\beta^{N}$	α^{+}/α, β^{0}/β^{N}	4	0.998
$\alpha\alpha/\alpha\alpha^{QS}$, β^{-28}/β^{N}	α^{+}/α, β^{+}/β^{N}	3	0.748
$\alpha\alpha/\alpha\alpha^{WS}$, β^{-28}/β^{N}	α^{+}/α, β^{+}/β^{N}	3	0.748
$\alpha\alpha/\alpha\alpha^{CS}$, β^{-28}/β^{N}	α^{+}/α, β^{+}/β^{N}	3	0.748
$\alpha\alpha/^{--SEA}$, $\beta^{CD71-72}/\beta^{N}$	α^{0}/α, β^{0}/β^{N}	3	0.748
$\alpha\alpha/\alpha\alpha^{WS}$, β^{CD17}/β^{N}	α^{+}/α, β^{0}/β^{N}	2	0.499
$\alpha\alpha/-\alpha^{4.2}$, $\beta^{CD14-15}/\beta^{N}$	α^{+}/α, β^{0}/β^{N}	2	0.499
$\alpha\alpha/^{--SEA}$, $\beta^{IVS-I-1}/\beta^{N}$	α^{0}/α, β^{0}/β^{N}	2	0.499
$\alpha\alpha/-\alpha^{3.7}$, $\beta^{IVS-I-1}/\beta^{N}$	α^{+}/α, β^{0}/β^{N}	2	0.499
$\alpha\alpha/^{--SEA}$, $\beta^{CD14-15}/\beta^{N}$	α^{0}/α, β^{0}/β^{N}	2	0.499
$-\alpha^{4.2}/--^{SEA}$, $\beta^{IVS-II-654}/\beta^{N}$	α^{+}/α^{0}, β^{+}/β^{N}	2	0.499
$\alpha\alpha^{WS}/^{--SEA}$, $\beta^{CD41-42}/\beta^{N}$	α^{+}/α^{0}, β^{0}/β^{N}	2	0.499
$-\alpha^{4.2}/--^{SEA}$, $\beta^{CD41-42}/\beta^{N}$	α^{+}/α^{0}, β^{0}/β^{N}	2	0.499
$\alpha\alpha/\alpha\alpha^{QS}$, $\beta^{IVS-II-654}/\beta^{N}$	α^{+}/α, β^{+}/β^{N}	1	0.249
$\alpha\alpha/\alpha\alpha^{CS}$, β^{CD26}/β^{N}	α^{+}/α, β^{E}/β^{N}	1	0.249
$\alpha\alpha/^{--SEA}$, β^{-29}/β^{N}	α^{0}/α, β^{+}/β^{N}	1	0.249
$\alpha\alpha/\alpha\alpha^{WS}$, $\beta^{CD71-72}/\beta^{N}$	α^{+}/α, β^{0}/β^{N}	1	0.249
$-\alpha^{3.7}/--^{SEA}$, $\beta^{CD41-42}/\beta^{N}$	α^{+}/α^{0}, β^{0}/β^{N}	1	0.249
$\alpha\alpha/\alpha\alpha^{WS}$, β^{-29}/β^{N}	α^{+}/α, β^{+}/β^{N}	1	0.249
$\alpha\alpha/-\alpha^{3.7}$, $\beta^{CD71-72}/\beta^{N}$	α^{+}/α, β^{0}/β^{N}	1	0.249
$\alpha\alpha/\alpha\alpha^{WS}$, β^{cap}/β^{N}	α^{+}/α, β^{+}/β^{N}	1	0.249
$\alpha\alpha/-\alpha^{4.2}$, β^{CD26}/β^{N}	α^{+}/α, β^{E}/β^{N}	1	0.249
$\alpha\alpha/^{--SEA}$, β^{CD43}/β^{N}	α^{+}/α, β^{0}/β^{N}	1	0.249
$\alpha\alpha/\alpha\alpha^{CS}$, $\beta^{IVS-II-654}/\beta^{N}$	α^{+}/α, β^{+}/β^{N}	1	0.249
$\alpha\alpha/-\alpha^{3.7}$, β^{cap}/β^{N}	α^{+}/α, β^{+}/β^{N}	1	0.249
$\alpha\alpha/\alpha\alpha^{WS}$, β^{CD26}/β^{N}	α^{+}/α, β^{E}/β^{N}	1	0.249
$\alpha\alpha/\alpha\alpha^{QS}$, $\beta^{CD41-42}/\beta^{N}$	α^{+}/α, β^{0}/β^{N}	1	0.249

$\alpha\alpha/\alpha\alpha^{CS}$, $\beta^{CD41\text{-}42}/\beta^{N}$	α^{+}/α, β^{0}/β^{N}	1	0.249
$\alpha\alpha/\alpha\alpha^{QS}$, β^{CD17}/β^{N}	α^{+}/α, β^{0}/β^{N}	1	0.249
$\alpha\alpha/\alpha\alpha^{CS}$, β^{CD17}/β^{N}	α^{+}/α, β^{0}/β^{N}	1	0.249
$-\alpha^{3.7}/-\alpha^{3.7}$, β^{CD17}/β^{N}	α^{+}/α^{+}, β^{0}/β^{N}	1	0.249
$-\alpha^{3.7}/--^{SEA}$, β^{-28}/β^{N}	α^{+}/α^{0}, β^{+}/β^{N}	1	0.249
$-\alpha^{4.2}/--^{SEA}$, $\beta^{IVS\text{-}I\text{-}1}/\beta^{N}$	α^{+}/α^{0}, β^{0}/β^{N}	1	0.249
$-\alpha^{3.7}/-\alpha^{4.2}$, β^{CD43}/β^{N}	α^{+}/α^{+}, β^{0}/β^{N}	1	0.249
$\alpha\alpha/\alpha\alpha^{WS}$, β^{CD26}/β^{N}	α^{+}/α, β^{E}/β^{N}	1	0.249
$\alpha\alpha/-\alpha^{HK}$, β^{-28}/β^{N}	α^{+}/α, β^{+}/β^{N}	1	0.249
$\alpha\alpha/\alpha\alpha^{WS}$,$\beta^{CD\ 15\ (TGG>TGA)}/\beta^{N}$	$\alpha+/\alpha$, β^{0}/β^{N}	1	0.249
Sum		401	100

Supplementary Data Table S5. Spectrum of δ- thalassemia, δβ- thalassemia and γ-thalassemia among people of reproductive age in Guangdong province, Southern China

genotype	Phenotype	HGVS Name	cases detected
$\delta^{-77\ T>C}/\delta^{CD87(CAG>TAG)}$	δ^{0}、δ^{+}/δ^{0}	HBD:c.-127T>C<br>HBD:c.262C>T	1
$\delta^{-30\ T>C}/\delta^{N}$	δ^{+}/δ^{N}	HBD:c.-80T>C	1
$\delta^{-77\ T>C}/\delta^{N}$	δ^{0}、δ^{+}/δ^{N}	HBD:c.-127T>C	2
δ-thal sum	-		4
Chinese $(A\gamma\delta\beta)^{0}$	$G\gamma(A\gamma\delta\beta)^{0}$	NC_000011.10:g.5169918_5248821del	16
δβ- thal sum	-		16
$\gamma^{-196\ C>T}/\gamma^{N}$	HPFH	HBG1:c.-249C>T	7
$\gamma^{-196\ C>T}/\gamma^{A\gamma(+25\ G>A)}$	HPFH<br>Hb F levels	HBG1:c.-249C>T<br>NG_000007.3:g.47783G>A	1
$\gamma^{-158\ C>T}\gamma/^{A\gamma(+25\ G>A)}$	Hb F levels<br>Hb F levels	NG_000007.3:g.47783G>A<br>NG_000007.3:g.42677C>T	1
$\gamma^{-219\ (+AGCA)}/\gamma^{N}$	HPFH	HBG1:c.-272_-275dup	1
γ-thal Sum	-		10

Supplementary Data Table S6. Spectrum and phenotypes of hemoglobinopathy among people of reproductive age in Guangdong province, Southern China

HGVS Name	Hemoglobin variantas	Phenotype	cases detected	Frequency (%)
HBA1:c.223 G>C	Hb-Q Thailand	HbVar	29	14.74
HBA1:c.205A>G	Hb Ube-2	HbVar	7	3.68
HBA1:c.84G>T	Hb Hekinan II	HbVar	4	2.11
HBA1:c.364G>A	Hb Owari	HbVar	2	1.05
HBA1:c.46G>C	Hb Ottawa	HbVar	2	1.05
HBA1:c.273G>T	Hb Guigang	HbVar	1	0.53
HBA2:c.80C>A	Hb Shenyang	HbVar	1	0.53
Partial Sum	-	-	46	23.68
HBA2:c.91 G>C	Hb G-Honolulu	HbVar	16	8.42
HBA2:c.34 A > C	Hb J-Wenchang-Wuming	HbVar	3	1.58
HBA2:c.77G>A	Hb Cibeles	HbVar	2	1.05
HBA2:c.245C>G	Hb Nigeria	HbVar	2	1.05
HBA2:c.146T>G	Hb Montgomery	HbVar	2	0.53
HBA2:c.51G>T	Hb Beijing	HbVar	1	0.53
HBA2:c.275T>C	Hb Port Phillip	HbVar	1	0.53
Partial Sum	-	-	26	14.21
HBB:c.341 T>A	Hb NewYork	HbVar	88	46.32
HBB:c.170G>A	Hb J-Bangkok	HbVar	13	6.84
HBB:c.68A>G	Hb G-Taipei	HbVar	7	3.68
HBB:c.68A>C	Hb G-Coushatta	HbVar	2	1.05
HBB:c.22G>A	Hb G-Siriraj	HbVar	2	1.05
HBB:c.233A>G	Hb Costa Rica	HbVar	1	0.53
HBB:c.238G>A	Hb Yaizu	HbVar	1	0.53
HBB:c.283G>C	Hb Barcelona	HbVar	1	0.53
Partial Sum	-	-	115	60.53
HBD:c.349C>T	Hb A2-Troodos	HbVar	2	1.05
HBD:c.27G>C	Hb A2-Hengyang	HbVar	1	0.53
Partial Sum	-	-	3	1.58
Sum	-	-	190	100

Supplementary Data Table S7. Allele frequency of α-thalassemia mutations among people of reproductive age in Guangdong province, Southern China.

**Mutation**	**HGVS name**	**Allele(n)**	**Allele Frequency (ratio%)**
$^{--SEA}$	NC_000016.10:g.165401_184701del	3866	46.96
-α3.7	NG_000006.1:g.34247_38050del	2322	28.20
-α4.2	NC_000016.10:g.169818_174075del	914	11.10
$\alpha\alpha^{WS}$	HBA2:c.369C>G	669	8.13
$\alpha\alpha^{CS}$	HBA2:c.427T>C	236	2.87
$\alpha\alpha^{QS}$	HBA2:c.377T>C	145	1.76
$\alpha\alpha^{HK}$	N/A	50	0.61
$^{--THAI}$	NC_000016.10:g.149863_183312del	15	0.18
Fusion gene/	N/A	1	0.01
$\alpha\alpha\alpha^{anti3.7}$	NG_000006.1:g.34247_38050dup	3	0.04
$\alpha\alpha\alpha^{anti4.2}$	NG_000006.1:g.(31957_31978)_(34525_34544)dup	4	0.05
$\alpha\alpha^{Init\ CD\ ATG>AAG}$	HBA2:c.2T>A,Initiation codon(ATG>AAG)	2	0.02
$\alpha\alpha^{CD\ 30\ -GAG}$	HBA2:c.91_93delGAG(CD 30 -GAG)	6	0.07
Sum		8233	100

Supplementary Data Table S8. Allele frequency of β-thalassemia mutations among people of reproductive age in Guangdong province, Southern China.

**Mutation**	**HGVS name**	**Allele(n)**	**Allele Frequency (ratio)**
$\beta^{\text{CD41-42}}$	HBB:c.126_129delCTTT	1022	39.84
$\beta^{\text{IVS-II-654}}$	HBB:c.316-197C>T	582	22.69
$\beta^{\text{-28}}$	HBB: c.-78A>G	394	15.36
β^{CD17}	HBB: c.52A>T	216	8.42
β^{CD26}	HBB: c.79G>A	104	4.05
$\beta^{\text{CD71-72}}$	HBB:c.217dupA	59	2.30
β^{cap}	HBB:c.-10_-7delAACA	36	1.40
$\beta^{\text{-29}}$	HBB: c.-79G>A	26	1.01
$\beta^{\text{CD27-28}}$	HBB:c.85dupC	25	0.97
β^{CD43}	HBB: c.130G>T	22	0.86
$\beta^{\text{IVS-I-1}}$	HBB: c.92 + 1G>T	18	0.74
$\beta^{\text{CD14-15}}$	HBB:c.45dupG	19	0.70
β^{Init}	HBB:c.1A>C	3	0.12
South East Asian (SEA) deletion	NC_000011.10:g.5201647_5229059del	14	0.55
Taiwanese deletion	NG_000007.3:g.69997_71353del1357	5	0.19
$\beta^{\text{-90 (C->T)}}$	HBB: c.-140C>T	2	0.08
$\beta^{\text{CD 89-93 (-14bp)}}$	HBB:c.268_281delAGTGAGCTGCACTG	2	0.08
$\beta^{\text{IVS-I(-2)}}$	HBB:c.91A>G	1	0.04
$\beta^{\text{CD8/9 (+G)}}$	HBB:c.27dupG	1	0.04
$\beta^{\text{CD43 (G->T)}}$	HBB: c.130G>T	1	0.04
$\beta^{\text{CD 15 (TGG>TGA)}}$	HBB:c.47G>A	1	0.04
$\beta^{\text{CD 37 (TGG>TAG)}}$	HBB:c.113G>A	6	0.23
$\beta^{\text{CD 19(AAC>AGC)}}$	HBB:c.59A>G	1	0.04
$\beta^{\text{IVS II-654 C>T}}$	HBB:c.316-197C>T	1	0.04
$\beta^{\text{IVS-I-110}}$	HBB:c.93-21G>A	1	0.04
$\beta^{\text{CD 27/28 (+C)}}$	HBB:c.85dupC	1	0.04
$\beta^{\text{-50 G>A}}$	HBB:c.-100G>A	1	0.04
deletion(4.903Kb)	chr11:5226187-5231090 deletion(4.903Kb)	1	0.04
total		2565	100
